# Sini Decoction Improves Adrenal Function and the Short-Term Outcome of Septic Rats through Downregulation of Adrenal Toll-Like Receptor 4 Expression

**DOI:** 10.1155/2018/5186158

**Published:** 2018-06-19

**Authors:** Fang Lai, Gengbiao Zhou, Shutao Mai, Xiaolian Qin, Wenting Liu, Yan Zhang, Dongping Xie, Yanna Weng, Jiongdong Du, Yi Zheng, Jiyang Liao, Yun Han

**Affiliations:** ^1^The Second Affiliated Hospital of Guangzhou University of Chinese Medicine, Guangzhou, Guangdong, China; ^2^Guangdong Provincial Hospital of Chinese Medicine, Guangzhou, Guangdong, China; ^3^Doctoral Student of the Second Clinical College of Guangzhou University of Chinese Medicine, Guangzhou, Guangdong, China; ^4^Guangdong Second Traditional Chinese Medicine Hospital, Guangzhou, Guangdong, China; ^5^Guixi Hospital of Traditional Chinese Medicine, Guixi, Jiangxi, China; ^6^Postgraduate Student of the Second Clinical College of Guangzhou University of Chinese Medicine, Guangzhou, Guangdong, China

## Abstract

**Background:**

Sini Decoction (SND) is composed of* Aconitum carmichaelii *Debeaux,* Zingiber officinale* Roscoe, and* Glycyrrhiza uralensis* Fisch, having been used in China for centuries for collapsing phrase of disease. Studies reported that SND could alleviate inflammatory response, ameliorate microcirculatory disturbances, and improve shock reversal and adrenal gland glucocorticoid stress response during sepsis shock, yet the underlying mechanism is still elusive. Toll-like receptor (TLR) 4 is demonstrated to be crucially correlated with the corticosterone secretion and the impaired adrenal glucocorticoid responses in sepsis.

**Materials and Methods:**

SND at dose of 10 g/kg (in low-dose SND group, LD-SND) and 20 g/kg (in high-dose SND group, HD-SND) was administered to CLP rats. Four days later, overall survival rates of rats were calculated; rat serum and adrenal glands were collected. Basic serum corticosterone levels were determined, and the increase of corticosterone after 0.8 ug/kg ACTH injection was checked to detect the adrenocortical sensitivity to ACTH. The protein and mRNA expression of TLR4 in adrenal glands were measured to study the impact of SND on TLR4 expression. mRNA levels of IL-10 and TNF-a in adrenal glands and IL-10 and TNF-a levels in serum were also determined to study the cytokines profile.

**Results:**

SND improved the cumulative survival rate of CLP rats up to 4 days (P < 0.05 with HD-SND) and adrenocortical sensitivity to 0.8 ug/kg ACTH stimulation (*P* < 0.05 at 60 mins, 31.02 ± 19.23 ng/ml in LD-SND group and 32.18 ± 14.88 ng/ml in HD-SND group versus 5.03 ± 13.34 ng/ml in CLP group), with a significant decrease of protein (P < 0.05, 29.6% in LD-SND group and 27.8% in HD-SND group), mRNA expression of TLR4 (P < 0.05, 32.9% in LD-SND group and 36.1% in HD-SND group), mRNA expression of IL-10 (P < 0.05, 32.0% in LD-SND group and 29.6% in HD-SND group), TNF-a in adrenal glands (P < 0.05, 26.0% in LD-SND group and 25.3% in HD-SND group), and TNF-a level in serum (P < 0.05, 100.20 ± 19.41 pg/ml in LD-SND group and 92.40 ± 11.66 pg/ml in HD-SND group versus 134.40 ± 27.87 pg/ml in CLP group).

**Conclusion:**

SND increased overall survival rate within 4 days and attenuated adrenal insufficiency in septic rats by downregulating TLR4 mRNA and protein expression in adrenal tissue, inhibiting adrenal production of TNF-*α* and IL-10, and improving adrenal responsiveness. Our results suggest that SND is able to ameliorate adrenal stress responses in a local immune-adrenal crosstalk way involving downregulated expression of TLR4 in adrenal tissue. SND might be a promising treatment for adrenal insufficiency prevention in prolonged sepsis.

## 1. Introduction

Sepsis, characterized by uncontrolled systemic inflammation probably causing life threatening organ dysfunction induced by infection, is still a worldwide major health problem with high incidence rates and mortality rates [1]. During sepsis, the hypothalamic-pituitary-adrenal axis (HPAA) is activated as a homeostasis-sustaining mechanism for maintaining vascular tone, endothelial integrity, and vascular permeability [2]. Absolute adrenal insufficiency (AAI), characterized by low cortisol baseline level and no response to ACTH stimulation, exists in some of the cases, especially in those with primary adrenal gland deficiency. But in most of the cases of sepsis, cortisol levels may increase. Yet the cortisol production might be still inadequate and far from enough sometimes, which is defined as “relative adrenal insufficiency” (RAI) [3, 4]. Clinical manifestations of RAI include refractory hypotension despite adequate fluid resuscitation and vasopressor therapy, inexplicable noninfective fever, and electrolytic abnormalities [5, 6]. Also, RAI occurs during the late stage of polymicrobial sepsis induced by CLP in rats [7, 8]. RAI is reported to be associated with increased mortality in sepsis [9–11]. Corticosteroid therapy might be an offer to benefit patients of sepsis with adrenal insufficiency [12, 13]. However, the lack of evidence for the underlying mechanism and a beneficial effect on mortality, not to mention the potential side effects of inducing gastroduodenal bleeding, superinfection, hyperglycaemia, hypernatraemia, neuromuscular weakness, etc., makes the corticosteroid therapy still a quite controversial issue nowadays [1, 14, 15].

Over the long history of human society, infectious diseases always take centre stage of fight with illness. Septic shock might be even more common dating back to the times when there was lack of effective modern medical technology. Traditional Chinese medicine has been used for thousands of years in China for multiple diseases including sepsis and septic shock, of which Sini Decoction (SND) is one of the most classical formulas, characterized by the essential effect of recuperating patients from collapse. SND was first recorded in* Shang Han Lun*, which is also called “Treatise on Exogenous Febrile Diseases”, the oldest monograph on infectious diseases written at the end of the Han dynasty dating back to 150-219 AD. And SND has been officially documented in China pharmacopoeia as a therapy for rescuing from collapse by restoring Yang and warming to dispel cold [16]. Studies reported that SND was effective in alleviating inflammatory response, ameliorating microcirculatory disturbances, and improving shock reversal and adrenal gland glucocorticoid stress response during sepsis shock, yet the underlying mechanism is still elusive [17–19].

Toll-like receptors (TLR) have been documented to be essential pattern-recognition receptors to initiate host defence against bacterial infections. They are documented to be expressed not only on immune cells but also on endocrine cells such as hypothalamus as well as pituitary and adrenal gland. Of the TLR, TLR4 is demonstrated as not only one of the most substantial receptors in activation of the HPAA during sepsis, but also a critical receptor to regulate the immune-adrenal crosstalk in sepsis [20, 21]. Stimulation of TLR4 on adrenocortical cells resulted in production of inflammatory factors including IL-8, TNF-*α*, and IL-6 in a dose-dependent way [22]. TLR4 also correlates with the corticosterone secretion and the impaired adrenal glucocorticoid responses in sepsis [20].

In the present study, we assessed the protective effects of SND on mortality and adrenal function in CLP septic rats. Furthermore, the mechanisms for these protective effects were explored by targeting TLR4.

## 2. Materials and Methods

### 2.1. Animals

Sprague-Dawley male rats (220-250 g) were purchased from Experimental Animal Center of Guangdong Province, housed in pathogen-free environment, and acclimated to a 12-hr light/dark cycle for five days. Rat chow and water were provided ad libitum until the time of surgery. All experiments were approved by the Ethics Committee of Experimental Animal of Guangdong Provincial Hospital of Chinese Medicine (permit number: 2016020) and complied with the regulations of “Guide for the Care and Use of Laboratory Animals” formulated by the National Institutes of Health.

### 2.2. Chemicals and Reagents

*Radix Aconiti Lateralis Preparata, Rhizoma Zingiberis, *and* Radix Glycyrrhizae *were offered by the pharmaceutical department of Chinese herbal medicine of Guangdong Provincial Hospital of Chinese Medicine. Anti-TLR4 antibody (Cat. ab30667) was supplied by Abcam (USA). Total RNA Kit II was provided by Omega (USA). PrimeScript™ RT reagent kit was purchased from Takara Biotechnology Co., Ltd. (Dalian, China). The DNA ladder/marker (25-500 bp) was obtained from Shanghai Sangon Biological Engineering Co., Ltd. (Shanghai, China). PVDF membranes were purchased from Millipore (MA, USA). Rat tumor necrosis factor- (TNF-) *α* and interleukin- (IL-) 10 enzyme-linked immunosorbent assay (ELISA) kits were offered by Dakewe Biotech Company (Shenzhen, China). Rat Cortisol ELISA kits were obtained from BioVision (California, USA). Rat ACTH ELISA Kits were purchased from Cusabio Biotech Company (Wuhan, China).

### 2.3. Preparation of Sini Decoction

*Radix Aconiti Lateralis Preparata, Rhizoma Zingiberis, *and* Radix Glycyrrhizae *(3:2:2) were immersed in distilled water (1 g: 20 ml) and boiled gently for 1 h twice. The two extracts were filtered, combined, condensed into 2 g/ml (the content of crude drug in the decoction, for the high-dose SND group) and 1 g/ml (for the low-dose SND group), respectively, and stored at 4°C before use.

### 2.4. Cecal Ligation and Puncture

The CLP surgery procedure was performed generally as follows: Rats were fasting 24 hours before surgery but allowed water ad libitum. Then they were anesthetized with an intraperitoneal dose of 3% pentobarbital (30 mg/kg body weight). A 3 cm midline laparotomy was performed after abdomens were shaved and disinfected to expose the cecum. The cecum was then gently isolated and exteriorized, and the membrane between the cecum and the mesentery was carefully cut to release the cecum. Then 25% of the cecum was ligated distally to the ileocecal valve with a 4-0 silk tie so as not to cause intestinal obstruction. Three punctures were made with an 16-gauge needle, and a small amount of the cecal contents was forced out of the cecum by squeezing. The ligated and punctured cecum was replaced into the abdominal cavity. The sham group animals underwent laparotomy without ligation or perforation. The anterior peritoneal wall and all skin incisions were closed in two layers with 3-0 silk sutures. The animals received an instant subcutaneous injection of 3 ml/100 g body weight normal saline for fluid resuscitation.

### 2.5. Grouping and Treatment

According to a random number table, 70 rats were randomly divided into 4 groups (n = 10 rats for the sham group, n = 20 rats for the rest groups): sham operation group (sham group), cecal ligation and puncture group (CLP group), high-dose Sini Decoction group (HD-SND group), and low-dose Sini Decoction group (LD-SND group). Sepsis was induced in the CLP and the two SND treatment groups by cecal ligation and puncture. For SND treatment groups, the rats received intragastric administration of SND (low dose: 10 g/kg, with a SND concentration of 1 g/ml; high dose: 20 g/kg, with a SND concentration of 2 g/ml) every day for 3 days from the day after operation. The sham group and the CLP group were administered with 10 ml/kg body weight of distilled water once a day instead (Table 1).

### 2.6. Tissue Collection and Blood Sampling

4 days after surgery, rats in each group were anesthetized by 3% pentobarbital (30 mg/kg body weight), and ACTH 0.8 ug/kg body weight was injected intraperitoneally to each rat. 0.5 ml of blood samples was taken from a catheter inserted into the right carotid artery right before and 30 mins after the injection of ACTH, respectively. And the rats were sacrificed 60 mins after ACTH injection. Blood was taken from the left ventricular cavity and abdominal aorta; plasma was isolated by centrifugation at 1600 g for 10 min. Adrenal gland tissues were removed from the abdominal cavity, washed with saline solution, dried with filter paper, and weighed. The plasma and tissues were stored at -80°C until needed for subsequent experiments. All procedures were done in groups of five to eight rats from 9 am to 12 am.

### 2.7. Survival Studies

All rats were allowed food and water ad libitum and monitored for 4 days after the surgery. The survival rates were evaluated daily for the 4-day period.

### 2.8. Determination of Serum Levels of TNF-a, IL-10, ACTH, and Corticosterone

Serum levels were measured by ELISA kits according to the manufacturer's instructions. The optical density was measured at a wavelength of 450 nm using a reference wavelength of 540 nm by automated enzyme-linked immunosorbent assay readers. Cytokines are expressed in pg/ml (TNF-a, IL-10, and ACTH) and ng/ml (corticosterone). All standards and samples were run in duplicate.

### 2.9. Western Blot of TLR4 Expression in Adrenal Glands

Adrenal glands were snap frozen in liquid nitrogen, pulverized, and resuspended in ice-cold lysis buffer (Solarbio, Beijing, China). Protein concentrations were determined with the BCA method. Lysates were allowed to solubilize on ice for 30 min, and particulate mass was removed by centrifugation at 15,000 x g for 15 min at 4°C. Samples were subjected to SDS-PAGE, and proteins were transferred to polyvinylidene fluoride (PVDF) membranes. Blots were blocked with 5% bovine serum albumin in TBS containing 0.5% Tween 20 (TBST) for 2 h and then incubated with an 1:1000 dilution of primary antibody for TLR4 overnight at 4°C on a rocker. After three times of 10 min washing with TBST, the blots were incubated with a 1:5000 dilution of HRP-conjugated species-specific respective secondary antibody for 1 h at room temperature. After another three times of 10 min washing with TBST, protein bands were visualized by chemiluminescence and then densitometric analysis was done by using Kodak IS4000R Imaging Station.

### 2.10. Quantitative Real-Time PCR Assay for RNAs of TLR4, TNF-a, and IL-10

Total RNA was extracted from the adrenal gland tissues of each group following the manufacturer's instructions of Total RNA Kit II (Omega, USA). RNA was reverse-transcribed to cDNA and mixed with SYBR Green PCR mastermix (TOYOBO, Osaka, Japan) and the primers listed in Table 2. The thermal cycling conditions were as follows: 30 cycles of 94°C for 30 s, 55°C for 30 s, and then 72°C for 1 min. GAPDH was used as the endogenous reference gene to normalize the data.

### 2.11. Statistical Analysis

Data from western blot and quantitative real-time PCR assay were normalized to control values and reported as percentage of the baseline values. Other numerical results were presented as mean ± SD. Levene's test was performed to assess the equality of variances between each group. The statistical significance of the above results was analyzed by one-way ANOVA followed by Tukey post hoc comparison, repeat measure of General Linear Model, or paired T test in time when appropriate. Significance of the differences between cumulative survival rates among groups was determined by log-rank test and the survival curve with the Kaplan-Meier method. SPSS (version 21.0, Chicago, IL, USA) for Windows was used for statistical analysis. Differences in values were considered significant if* P* < 0.05.

## 3. Results

### 3.1. HD-SND Improved Survival Rates of CLP Induced Sepsis Rats

**Figure 1:**
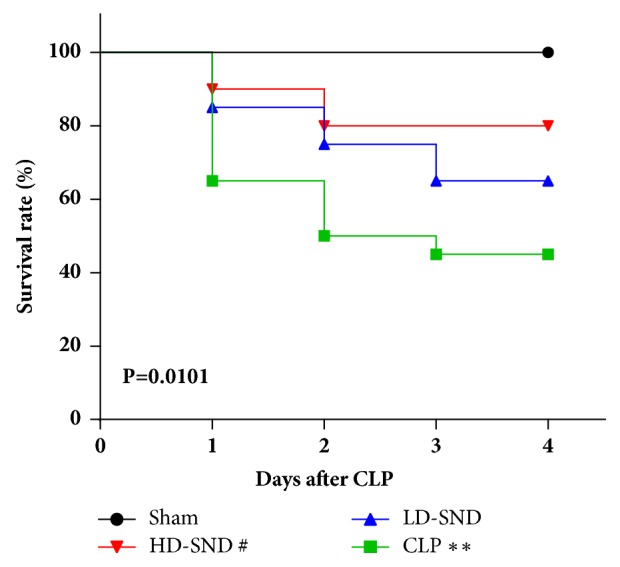
**Survival rates between 4 groups** (n = 10 for the sham group, n = 20 for the rest three groups) within 4 days. *∗∗P* < 0.01 versus sham group; #*P* < 0.05 versus CLP group.

The cumulative survival rates up to 4 days after surgery of different groups are shown in Figure 1, with a 4-day postoperative survival rate of 100% for the sham group, 80% for the HD-SND group, 65% for the LD-SND group, and 45% for the CLP group. The cumulative survival rates of the sham and HD-SND groups (P < 0.01 and P < 0.05, respectively) were significantly higher than the CLP group, while no significant differences were identified between the LD-SND group and the CLP group (P > 0.05).

### 3.2. SND Improved Adrenocortical Responsiveness to Exogenous ACTH

**Figure 2:**
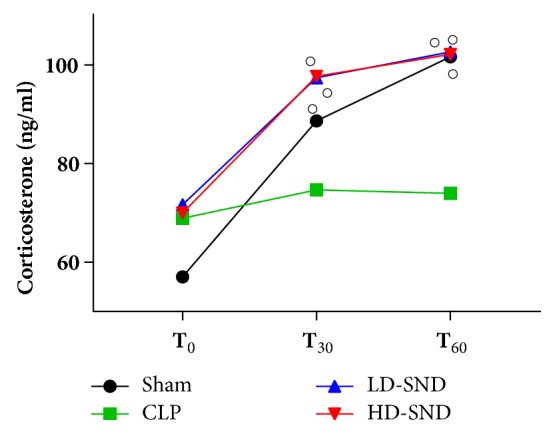
**Comparison of plasma corticosterone from the time before ACTH stimulation to 60 mins after ACTH stimulation between the four groups** (n = 7 per group,* P* = 0.58 by Repeated Measures of General Linear Model). ○*P* < 0.05 versus T_0_ values of the respective group (by paired T test).

**Figure 3:**
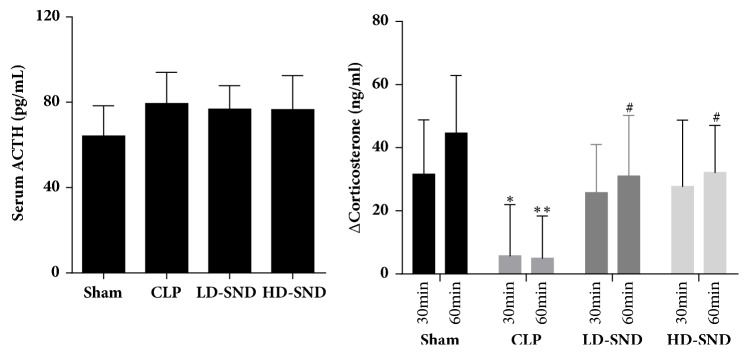
**Comparison of plasma ACTH (n = 7 per group) and the increase of serum corticosterone (n = 7 per group) 30 mins and 60 mins after ACTH stimulation between the four groups, all comparing to other groups of the respective time**. *∗P* < 0.05 versus sham group; *∗∗P* < 0.01 versus sham group; #*P* < 0.05 versus CLP group.

There was no significant difference in plasma level of corticotropin and corticosterone between the four groups before exogenous corticotropin injection (P > 0.05). After 0.8 ug/kg ACTH injection into the abdominal cavity, similar trends were found in corticosterone of the sham, LD-SND, and HD-SND groups, increasing significantly at 30 mins and 60 mins compared to the baseline of the respective group (*P* < 0.05). In contrast, the CLP group showed a blunted corticosterone secretion in response to ACTH stimulation (P > 0.05) (Figure 2). Compared to the CLP group, significant differences were found in the increase of corticosterone in the sham group (*P* < 0.05 at 30 mins,* P* < 0.01 at 60 mins), the LD-SND group (*P* < 0.05 at 60 mins), and the HD-SND group (*P* < 0.05 at 60 mins) (Figure 3).

### 3.3. SND Suppressed TLR4 Expression in Adrenal Tissues

**Figure 4:**
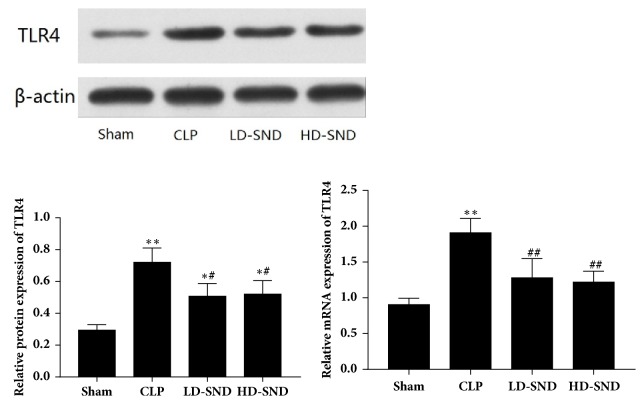
**TLR4 protein and mRNA expression in adrenal tissue 4 days after CLP** (n = 4 per group). *∗P* < 0.05 versus sham group; *∗∗P* < 0.01 versus sham group; #*P* < 0.05 versus CLP group; ##*P* < 0.01 versus CLP group.

The protein and mRNA expression of TLR4 in rat adrenal glands significantly increased in CLP group comparing to the sham group (P < 0.01). However, the protein and mRNA expression of TLR4 significantly decreased with SND therapy comparing with the CLP group (P < 0.05) (Figure 4).

### 3.4. SND Decreased TNF-*α* and IL-10 Levels in the Adrenal Glands

**Figure 5:**
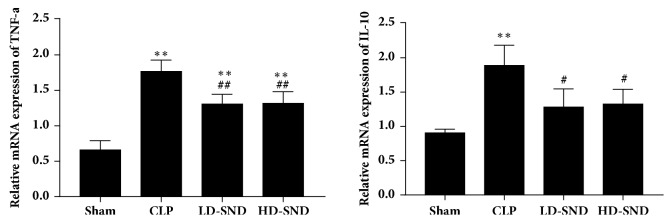
**TNF-a and IL-10 mRNA expression in adrenal tissue 4 days after CLP** (n = 4 per group), *∗∗P* < 0.01 versus sham group; #*P* < 0.05 versus CLP group; ##*P* < 0.01 versus CLP group.

As shown in Figure 5, CLP procedure led to significant increases in mRNA levels of IL-10 and TNF-a. Of note, CLP induced alterations of cytokines were remarkably suppressed by SND therapy at the transcriptional levels in adrenal glands (Figure 5).

### 3.5. SND Decreased Plasma TNF-a Levels

**Figure 6:**
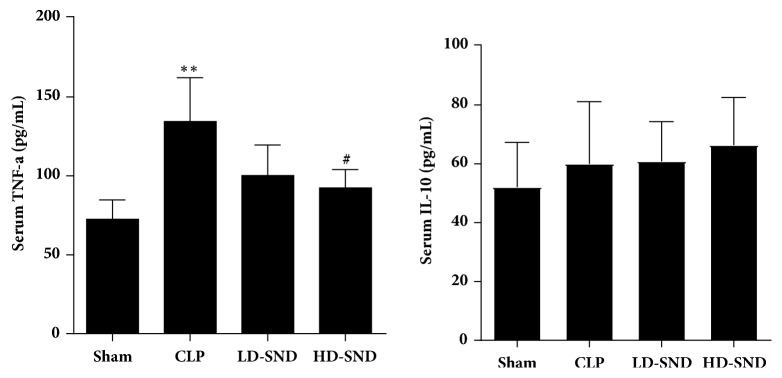
**Comparison of the mean levels of IL-10 and TNF-a in the plasma of rats with different treatment**. Data are presented in pg/mL as the mean ± standard deviation (n = 5 per group). *∗∗P* < 0.01 versus sham group; #*P* < 0.05 versus CLP group.

Compared to the sham group, significant increase of the plasma TNF-a level was seen in the CLP group (P < 0.01, Figure 6). Compared to the CLP group, the plasma TNF-a level was dramatically reduced with SND therapy, and significant differences were found between the HD-SND group and the CLP group (P < 0.05). However, there were no significant differences in plasma IL-10 levels among all four groups (Figure 6).

## 4. Discussion

The results of the present study provided the first evidence of SND that protects against sepsis and sepsis-induced adrenal dysfunction in rat model by downregulation of TLR4. The main findings of this study were that SND could improve short-term survival rates, attenuate adrenal stress responses, and downregulate local TLR4, TNF-*α*, and IL-10 expression in adrenal tissue induced by CLP. These results suggested that SND may be a potential protective therapy for sepsis.

CLP model is a typical self-infection model of induced sepsis by local abdominal infection originated from the leaked cecum after surgery of ligation and puncture on the cecum. Due to the simplification and the high-degree similarity to human sepsis of the operation, CLP model has been considered as the gold standard animal model in sepsis study universally [23, 24]. Improvement of survival is always regarded as the most solid and direct judgment for sepsis therapy. In the present study, the mortality rate of the CLP group within 4 days was 55%, and with the SND treatments the survival rates could be improved, demonstrating the efficacy of SND therapy to protect lethal sepsis in CLP rats.

Sepsis causes systemic inflammation-related dysregulation of not only the lung, kidney, heart, etc. but also the HPAA [20]. When there is no stress, cortisol is secreted in a diurnal pattern. But when there is a great stress, severe sepsis, for example, the diurnal rhythm disappears. Inflammatory cytokines, such as IL-6 and TNF-*α*, can stimulate the HPAA both in physiological and in pathological ways. Physiologically, adequate amount of inflammatory cytokines stimulates the hypothalamus to release corticotrophin releasing factor (CRF), which promotes the anterior pituitary gland to produce ACTH; ACTH activates the adrenal cortex to produce glucocorticoids and other steroids. At the same time, glucocorticoids inhibit the HPAA through a feedback mechanism so as to maintain a dynamic balance in neuroendocrine-immune regulation [5]. Pathologically, for example, in sepsis, the sudden outbursts of inflammatory cytokines stimulate the HPAA to increase secretion of cortisol and lose its circadian rhythm as a crucial adaptation component to maintain homeostasis in a stress situation [25]. And it has been documented that the dysregulation of HPAA also occurs in the late stage of polymicrobial sepsis in CLP rats [7, 8]. In the present study, the sham group rats showed a relatively normal level of corticosterone with a sound response to ACTH stimulation, while the CLP rats showed adrenal dysfunctions 4 days after surgery including increased baseline plasma corticosterone and suppression of adrenocortical sensitivity to ACTH. The above-mentioned results are consistent with the documented changes in experimental study about adrenal function in sepsis at 4 days [8]. And with SND therapy the suppressed adrenocortical sensitivity to ACTH is progressively improved.

And it has also been reported that corticosterone secretion and adrenocortical responsiveness to ACTH in the pm hours (1630~1800 hrs) were greater than in the am hours (0800~1000 hrs) in the sham group compared to the CLP group, while the CLP rats lost the diurnal rhythm in cortisol and showed no pm to am difference within 1 week after CLP procedure [8]. In the present study, all the blood samples of corticosterone were taken during the forenoon, so the significance of difference in corticosterone between groups may be less obvious according to study of Carlson et al. Nevertheless, the differences between SND groups and the CLP group still showed statistical significance.

Data indicate that TLR are critical receptors mediating inflammatory responses to infection. TLR4 is one of the TLR families and is involved in the recognition of lipopolysaccharide (LPS), innate immunity, and inflammation signaling [26]. And TLR4 is also found to play a crucial role in immune-adrenal crosstalk, modulating adrenal glucocorticoid stress response during infections [20, 21, 26]. The HPAA function impairs at the adrenal level due to the systematic absence of TLR4 [21]. But TLR4 is involved not only in a pituitary-dependent way. The fact that many critical ill patients maintain normal or even elevated level of glucocorticoids while the CRH and ACTH levels are suppressed suggests the adrenal stress response regulation of neuroendocrine circuits prevails by the regulation of the local intra-adrenal regulation [27], especially in late sepsis [20]. Structurally, TLR4 is found to be expressed in adrenal cells, both in human and in rats [26, 28]. Furthermore, direct stimulation with TLR4 ligands on human adrenocortical cell can cause a dose-dependent secretion of cortisol [29]. Meanwhile, TLR4 overexpression in human adrenocortical cells directly leads to impairment of cortisol and aldosterone production [30]. In the present study, TLR4 protein and mRNA expression in adrenal tissue were significantly decreased with SND therapy comparing with the CLP group, with an improvement in adrenal responsiveness and survival rates in SND groups. It seemed that SND therapy helped the adrenal TLR4 to stay at relatively appropriate levels and helped the adrenal stress systems to remain in competent coordinated response, which might be crucial for survival in sepsis.

It is documented that adrenal gland acts not only as the action site of numerous cytokines, but also as the synthesis site of cytokines [26]. Stimulation of TLR4 on adrenocortical cells could result in induction of TNF-*α*, IL-6, and IL-8 in a dose-independent way [22]. In the present study, along with the downregulation of TLR4 expression in the adrenal glands, the mRNA expression of TNF-*α* and IL-10, the typical representative of proinflammatory cytokines and anti-inflammatory cytokines, respectively, was also downregulated. And it has been reported that the levels of TNF-*α* in adrenal tissue of CLP mice are associated with the severity of adrenal inflammation, adrenal incapacitation, and mortality [31], which are in accordance with the results of the present study. And the serum TNF-*α* levels were decreased with SND therapy in the present study, indicating the probability of systematic inflammation alleviation, in accord with the result of Xu et al. study [17].

It is worth noting that the underlying mechanism of adrenal TLR4 expression downregulation by SND is still elusive. And also we are conscious of the limitations of the present study performed solely in rats. Further studies involving human tissues on underlying mechanism are required.

## 5. Conclusion

The results of this study indicate that relative adrenal dysfunction as indicated by interrupted circadian rhythm corticosterone secretion and suppressed responses to exogenous corticotropin occurs during the late stage of polymicrobial sepsis. And 3-day administration of SND raised the net increase of plasma corticosterone in plasma after exogenous corticotropin stimulation and improved 4-day mortality of CLP rats. The mechanism of these SND protective effects on sepsis may be through downregulation of adrenal TLR4 expression. Therefore, SND might be a potential therapy to improve adrenal responsiveness and the outcome of sepsis.

## Figures and Tables

**Table 1:** Oral administration of medicine was conducted once a day for 3 days, starting from the day after induction of CLP.

**Group**	**N**	**Operation**	**Oral administration**
Sham	10	Sham	Distilled water (10ml/kg)
CLP	20	CLP	Distilled water (10ml/kg)
LD-SND	20	CLP	Low dose of SND (10g/kg)
HD-SND	20	CLP	High dose of SND(20g/kg)

**Table 2:** Primers list for real-time PCR analysis.

Gene	Primer	Sequence (5′~3′)
TLR4	F	GGGGCAGTAGTAGGTGCTCAA
	R	GCCTGTGTTCAGTTTCCATTC
TNF-a	F	GCGCCCAGCCTTCCTTAC
	R	GCCCCGGCCTTCCAAATAAATAC
IL-10	F	TTGAACCACCCGGCATCTAC
	R	CCAAGGAGTTGCTCCCGTTA
GAPDH	F	GATGCAGAAGGAGATCACTGC
	R	ATACTCCTGCTTGCTGATCCA

F: forward primer; R: reverse primer.

## Data Availability

The datasets used and analyzed during the current study are available from the corresponding author on reasonable request.

## Supplementary Materials

Supplementary Materials Chemical profiling of main constituents in SND, using ultrahigh pressure liquid chromatography coupled with LTQ Orbitrap mass spectrometry. 17 main constituents were profiled by comparing the mass information with those reported in literatures [32, 33].

## References

[1] Rhodes A., Evans L. E., Alhazzani W. (2017). Surviving sepsis campaign: international guidelines for management of sepsis and septic shock: 2016. *Critical Care Medicine*.

[2] Lamberts S. W. J., Bruining H. A., De Jong F. H. (1997). Corticosteroid therapy in severe illness. *The New England Journal of Medicine*.

[3] Elbuken G., Karaca Z., Tanriverdi F. (2016). Comparison of total, salivary and calculated free cortisol levels in patients with severe sepsis. *Journal of Intensive Care*.

[4] Kertai M. D., Fontes M. L. (2015). Predicting adrenal insufficiency in severe sepsis: The role of plasma-free cortisol. *Critical Care Medicine*.

[5] Zaloga G. P., Marik P. (2001). Hypothalamic-pituitary-adrenal insufficiency. *Critical Care Clinics*.

[6] Lipiner-Friedman D., Sprung C. L., Laterre P. F. (2007). Adrenal function in sepsis: The retrospective Corticus cohort study. *Critical Care Medicine*.

[7] Koo D. J., Jackman D., Chaudry I. H., Wang P. (2001). Adrenal insufficiency during the late stage of polymicrobial sepsis. *Critical Care Medicine*.

[8] Carlson D. E., Chiu W. C., Scalea T. M. (2006). Cecal ligation and puncture in rats interrupts the circadian rhythms of corticosterone and adrenocortical responsiveness to adrenocorticotrophic hormone. *Critical Care Medicine*.

[9] De Jong M. F. C., Beishuizen A., Van Schijndel R. J. M. S., Girbes A. R. J., Groeneveld A. B. J. (2012). Risk factors and outcome of changes in adrenal response to ACTH in the course of critical illness. *Journal of Intensive Care Medicine*.

[10] Lim S. Y., Kwon Y. S., Park M. R. (2011). Prognostic significance of different subgroup classifications of critical illness-related corticosteroid insufficiency in patients with septic shock. *Shock*.

[11] De Jong M. F. C., Beishuizen A., Spijkstra J.-J., Groeneveld A. B. J. (2007). Relative adrenal insufficiency as a predictor of disease severity, mortality, and beneficial effects of corticosteroid treatment in septic shock. *Critical Care Medicine*.

[12] Katsenos C. S., Antonopoulou A. N., Apostolidou E. N. (2014). Early administration of hydrocortisone replacement after the advent of septic shock: impact on survival and immune response. *Critical Care Medicine*.

[13] Annane D., Sebille V., Charpentier C. (2002). Effect of treatment with low doses of hydrocortisone and fludrocortisone on mortality in patients with septic shock. *JAMA*.

[14] Keh D., Trips E., Marx G. (2016). Effect of Hydrocortisone on Development of Shock Among Patients With Severe Sepsis. *Journal of the American Medical Association*.

[15] Volbeda M., Wetterslev J., Gluud C., Zijlstra J. G., van der Horst I. C. C., Keus F. (2015). Glucocorticosteroids for sepsis: systematic review with meta-analysis and trial sequential analysis. *Intensive Care Medicine*.

[16] National Pharmacopoeia Council (2015). *Pharmacopoeia of the People’s Republic of China*.

[17] Xu M. J., Huang R. L., Chang X., Qiao Q. J., Zhang Z., Wang L. (2013). The effect of Sini Decoction on inflammatory cytokines in Sepsis Shock. *Traditional Chinese Medicine Journal*.

[18] Huang R. L., Zhang Z., Xu M. J. (2014). Effect of Sini decoction on function of hypothalamic-pituitary-adrenal axis in patients with sepsis. *Chinese Critical Care Medicine*.

[19] Zhang H., Sugiura Y., Wakiya Y., Goto Y. (1999). Sinitang (Shigyaku-to), a traditional Chinese medicine improves microcirculatory disturbances induced by endotoxin in rats. *Journal of Ethnopharmacology*.

[20] Kanczkowski W., Alexaki V.-I., Tran N. (2013). Hypothalamo-pituitary and immune-dependent adrenal regulation during systemic inflammation. *Proceedings of the National Acadamy of Sciences of the United States of America*.

[21] Zacharowski K., Zacharowski P. A., Koch A. (2006). Toll-like receptor 4 plays a crucial role in the immune-adrenal response to systemic inflammatory response syndrome. *Proceedings of the National Acadamy of Sciences of the United States of America*.

[22] Kanczkowski W., Zacharowski K., Wirth M. P., Ehrhart-Bornstein M., Bornstein S. R. (2009). Differential expression and action of Toll-like receptors in human adrenocortical cells. *Molecular and Cellular Endocrinology*.

[23] Dejager L., Pinheiro I., Dejonckheere E., Libert C. (2011). Cecal ligation and puncture: the gold standard model for polymicrobial sepsis?. *Trends in Microbiology*.

[24] Hubbard W. J., Choudhry M., Schwacha M. G. (2005). Cecal ligation and puncture. *Shock*.

[25] Marik P. E., Zaloga G. P. (2002). Adrenal insufficiency in the critically III: A new look at an old problem. *CHEST*.

[26] Bornstein S. R., Ziegler C. G., Krug A. W. (2006). The role of toll-like receptors in the immune-adrenal crosstalk. *Annals of the New York Academy of Sciences*.

[27] Kanczkowski W., Sue M., Bornstein S. R. (2016). Adrenal gland microenvironment and its involvement in the regulation of stress-induced hormone secretion during sepsis. *Frontiers in Endocrinology*.

[28] Bornstein S. R., Schumann R. R., Rettori V., McCann S. M., Zacharowski K. (2004). Toll-like receptor 2 and toll-like receptor 4 expression in human adrenals. *Hormone and Metabolic Research*.

[29] Vakharia K., Hinson J. P. (2005). Lipopolysaccharide directly stimulates cortisol secretion by human adrenal cells by a cyclooxygenase-dependent mechanism. *Endocrinology*.

[30] Kanczkowski W., Tymoszuk P., Chavakis T. (2011). Upregulation of TLR2 and TLR4 in the human adrenocortical cells differentially modulates adrenal steroidogenesis. *Molecular and Cellular Endocrinology*.

[31] Jennewein C., Tran N., Kanczkowski W. (2016). Mortality of Septic Mice Strongly Correlates with Adrenal Gland Inflammation. *Critical Care Medicine*.

[32] Montoro P., Maldini M., Russo M., Postorino S., Piacente S., Pizza C. (2011). Metabolic profiling of roots of liquorice (*Glycyrrhiza glabra*) from different geographical areas by ESI/MS/MS and determination of major metabolites by LC-ESI/MS and LC-ESI/MS/MS. *Journal of Pharmaceutical and Biomedical Analysis*.

[33] Sun H., Ni B., Zhang A., Wang M., Dong H., Wang X. (2012). Metabolomics study on Fuzi and its processed products using ultra-performance liquid-chromatography/electrospray-ionization synapt high-definition mass spectrometry coupled with pattern recognition analysis. *Analyst*.

