# Supplementary material for: Sini Decoction Improves Adrenal Function and the Short-Term Outcome of Septic Rats through Downregulation of Adrenal Toll-Like Receptor 4 Expression
Source: Evid Based Complement Alternat Med. 2018 Jun 19;2018:5186158. doi: 10.1155/2018/5186158 (PMC6029449; doi:10.1155/2018/5186158)
Supplement: Supplementary Materials — Chemical profiling of main constituents in SND, using ultrahigh pressure liquid chromatography coupled with LTQ Orbitrap mass spectrometry. 17 main constituents were profiled by comparing the mass information with those reported in literatures [32, 33]. [file 5186158.f1.zip › supplementary material_ECAM_2350749.pdf]

Supplementary Material

Chemical profiling of main constituents in SND, using ultra-high pressure liquid chromatography coupled with LTQ Orbitrap mass spectrometry

1 Chemicals and standards. HPLC grade methanol was purchased from Fisher Chemicals (Fairlawn, NJ, USA). Formic acid was AR grade and purchased from Guangzhou Chemical Reagent Corporation (Guangzhou, China). Water used in the experiment was deionized and further purified by a Milli-Q Plus water purification system (Millipore Ltd. USA). Other reagents and chemicals were of analytical grade. Benzoylmesaconine, Benzoylaconitine, Liquiritin, Liquiritin apioside, Isoliquiritin and Glycyrrhizic acid were kindly offered by Dr Xiong Li (Guangdong Provincial Academy of Chinese Medical Sciences).

2 SND samples preparation. 5 ml sample (Sample 1-1: high-dose SND, with a SND concentration of 2g/ml; Sample 1-2: low dose SND, with a SND concentration of 1g/ml) was added MeOH to 50 ml, and the solution was filtered and concentrated under 60°C by reduced pressure using a rotary evaporator, then dissolved by 5 ml 50%MeOH. 1-1 was further diluted 5 times before analysis, while 1-2 was directly analyzed. The solution was filtered through a 0.22-μm nylon membrane filter prior to injection for LC/MS analysis.

3 UHPLC-PAD/ESI-MSn analysis. Chromatographic separation was performed on an Accela^{TM} U-HPLC system (Thermo Fisher Scientific, San Jose, CA, USA) comprising a U-HPLC pump, a PDA detector, scanning from 200 to 400 nm. The LC conditions were as follows: Inertsil ODS-3 column, 100 × 2.1 mm i.d., 3 µm; Mobile phase: (A) MeOH; (B) H_2O with 0.1% formic acid; Flow rate: 350 µL/min; Injection volume: 5-10 µL; Gradient: 0-3 min, 15-30 %A; 3-9 min, 30-31% A; 9-11 min, 31-32% A; 11-15 min, 32-40% A; 15-20 min, 40-42% A; 20-35 min, 42-60% A; 11-15 min, 32-40% A; 35-40 min, 60-100% A; 40-50 min, 15 A.

MS analysis was performed using a LTQ Orbitrap XL hybrid mass spectrometer (Thermo Fisher Scientific, San Jose, CA, USA), fitted with an ESI source, and operated in positive ion mode, with a mass range of 125–1000 with resolution set at 30,000 using the normal scan rate. The non-target data-dependent MS/MS scan mode was performed on the most intense ions detected in full scan. The MS/MS isolation width was 1 amu, and the normalized collision energy was 30% for all compounds. Nitrogen was used as sheath gas and helium served as the collision gas. The electrospray parameters were as follows: source voltage: 4.0 kV; sheath gas (nitrogen): 45 L/min; auxiliary gas flow: 5 L/min; capillary voltage: 35.0 V; capillary temperature: 325.0 ◦C; MS scan functions and HPLC solvent gradients were controlled by the Xcalibur data system (Thermo Fisher, San Jose, CA, USA). Data was collected and analyzed

with Xcalibur 2.07 (Thermo Scientific). The Orbitrap mass analyzer was calibrated according to the manufacturer's directions using a commercial calibration fluid.

4 Characterization of main constituents in SND. Chromatographic separation was performed by PDA total scan (Figure 7). A qualitative analysis was carried out in positive ionization mode and accurate mass data was acquired in the full scan analysis, and the production mass was obtained in the data-dependent MS scan (Figure 8). 17 main constituents (Table **3**) were profiled by comparing the mass information with those reported in literatures [32, 33]. Benzoylmesaconine (**3**), Benzoylaconitine (**4**), Liquiritin (**6**), Liquiritin apioside (**7**), Isoliquiritin (**10**) and Glycyrrhizic acid (**17**) were definitely identified by comparing the retention time with those of standards (Figure 9). As we can see from Figure 7 and Table 3, the main componets profiled in the SND are flavonoids from Glycyrrhiza glabra, and alkoloids from Fuzi, while the alkoloids idnetified in Fuzi are main monoester diterpene alkaloids with less or non-toxic.

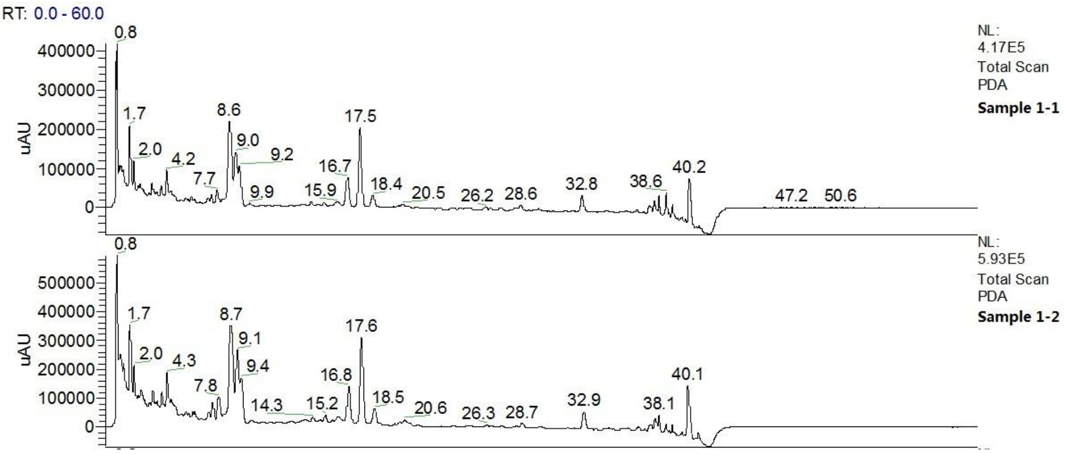

Figure 7 PDA total scan of Sample 1-1 and 1-2.

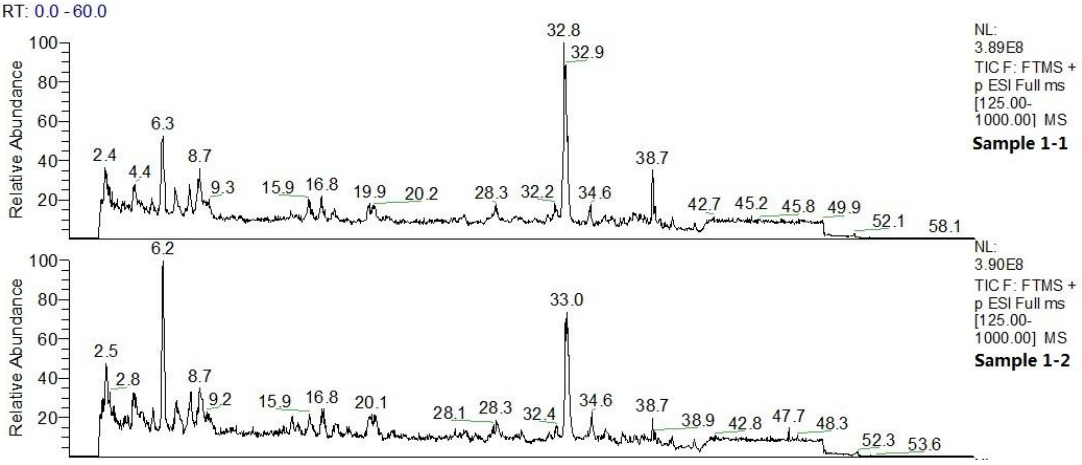

Figure 8 Full scan analysis in positive ionization mode of Sample 1-1 and 1-2.

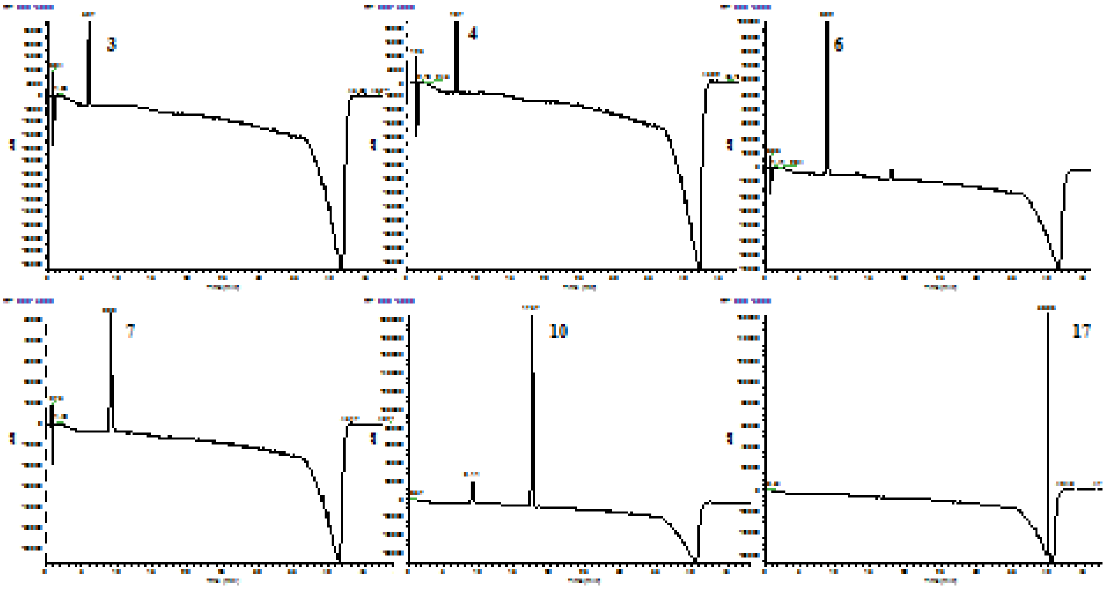

Figure 9 Standard retention time of Benzoylmesaconine (3), Benzoylaconitine (4), Liquiritin (6), Liquiritin apioside (7), Isoliquiritin(10) and Glycyrrhizic acid (17).

Table 3: 17 main constituents profiled by reported literatures.

No.	TR(min)	$[M+H]^+$	Fomula	ppm	MS2 product ions	Identification
1	4.2	598.21191	C27H36O14N	-1.87	419.13351(100), 257.08099(88)	Unknown alkoloids
2	4.3	228.08649	C10H14O5N	-0.70	107.04892(100), 211.06027(12), 172.04253(12)	Unknown alkoloids
3	6.2	590.29413	C31H44O10N	-3.55	540.25885(100), 558.26959(45), 508.23297(25), 572.28540(22), 526.24365(15)	Benzoylmesaconine
4	7.1	604.30988	C32H46O10N	-2.19	554.27441(100), 572.28510(36), 586.30096(18), 522.24860(17), 540.25922(11)	Benzoylaconitine
5	8	574.29974	C31H44O9N	-3.13	542.27441(100), 510.24872(7)	Benzoylhypacoitine
6	8.6	436.15945	C21H22O9	-1.58	257.08096(100)	liquiritin
7	9	551.1745	C26H30O13	-2.57	257.08105(100)	Liquiritin/Isoliquiritin apioside
8	9.3	551.17468	C26H30O13	-2.24	257.08105(100)	Liquiritin/Isoliquiritin apioside
9	16.8	431.13275	C22H23O9	-1.83	269.08087(100)	Ononin
10	17.7	419.13266	C21H22O9	-2.03	257.0809(100), 258.08441(6)	Isoliquiritin
11	32.8	312.21671	C17H30O4N	-0.72	277.17953(100)	Unknown
12	36.7	357.16904	C21H24O5	-1.71	301.10718(100), 235.13303(64), 221.11729(98)	Unknown
13	37.9	355.11713	C20H18O6	-1.28	336.09506(100), 189.09111(97)	Unknown
14	38.2	369.13272	C21H21O6	-1.40	313.07068(100)	Glycycoumarin
15	38.7	277.17938	C17H25O3	-1.59	137.05956(100), 235.16931(34), 259.20575 (15)	Unknown
16	40	279.15848	C16H23O4	-2.17	149.02316(100), 205.08597(10)	Unknown
17	40.2	823.40863	C42H63O16	-2.95	—	Glycyrrhizic acid
